# Comparison of the Anticancer Effects of Arvanil and Olvanil When Combined with Cisplatin and Mitoxantrone in Various Melanoma Cell Lines—An Isobolographic Analysis

**DOI:** 10.3390/ijms232214192

**Published:** 2022-11-16

**Authors:** Paweł Marzęda, Paula Wróblewska-Łuczka, Magdalena Florek-Łuszczki, Małgorzata Drozd, Agnieszka Góralczyk, Jarogniew J. Łuszczki

**Affiliations:** 1 Department of Occupational Medicine, Medical University of Lublin, 20-090 Lublin, Poland; 2 Department of Medical Anthropology, Institute of Rural Health, 20-950 Lublin, Poland

**Keywords:** cannabinoids, melanoma, arvanil, olvanil, drug interactions, in vitro

## Abstract

Due to the unique structures of arvanil and olvanil, the drugs combine certain properties of both cannabinoids and vanilloids, which makes them able to stimulate both TPRV1 and CB1 receptors and causes them to be interesting agents in the setting of carcinoma treatment. The aim of this study was to investigate the cytotoxic and anti-proliferative effects of arvanil and olvanil when administered alone and in combination with cisplatin (CDDP) and mitoxantrone (MTX), using various primary (A375, FM55P) and metastatic (SK–MEL 28, FM55M2) human malignant melanoma cell lines. The results indicate that both arvanil and olvanil inhibited (dose-dependently) the viability and proliferation of various malignant melanoma cells, as demonstrated by MTT and BrdU assays. The safety profile of both arvanil and olvanil tested in human keratinocytes (HaCaT) and normal human melanocytes (HEMa–LP) revealed that neither arvanil nor olvanil caused significant cytotoxicity in HaCaT and HEMa–LP cell lines in LDH and MTT assays. Isobolographically, it was found that both arvanil and olvanil exerted additive interactions with MTX and antagonistic interactions with CDDP in the studied malignant melanoma cell lines. In conclusion, the combinations of arvanil or olvanil with MTX may be considered as a part of melanoma multi-drug therapy; however, the combination of these compounds with CDDP should be carefully considered due to the antagonistic interactions observed in the studied malignant melanoma cell lines.

## 1. Introduction

Cutaneous melanoma is a malignant neoplasm deriving from melanin-producing cells. In 2020, the standardized incidence rate of melanoma reached 3.0 in women and 3.8 in men, which represents 324,625 new melanoma cases and 1.7% of total new cases of the most common cancers [1]. The main risk factor is exposure to ultraviolet radiation. People with certain phenotypes have a predilection to develop melanoma, including those with a pale complexion, light-coloured eyes, and freckles. The other risk factors are immunosuppression and genetic predispositions, such as CDKN2 gene mutation and xeroderma pigmentosum [2]. The primary prophylaxis is protection against excessive ultraviolet radiation. The positive impact of the growth of public awareness and using sun-protection has already been noticed in the incidence rates of melanoma in Australia [1].

The treatment of melanoma in the early stages include wide excision of nevus, with lymph node dissection and stereotactic irradiation if necessary; in the metastatic stage, the treatment is systemic therapy above all [3]. After nearly 30 years of increasing melanoma mortality rates, a significant decrease by 17.9% has been observed since 2013 due to the introduction of novel therapies in metastatic melanoma, including anti PD-1 and anti CTLA-4 antibodies [4]. However, the 5-year survival rates still remain low in regional (68%) and in distant stages (30%); therefore, more studies on treatment of melanoma are required [5].

Cannabinoids together with their two main receptors, the CB1 and CB2 receptors, have recently gained attention as potential anticancer agents. Similar interest has been placed in the determination of the antineoplastic properties of transient receptor potential cation channel subfamily V (TRPV) members, especially TRPV1.

**Figure 1:**
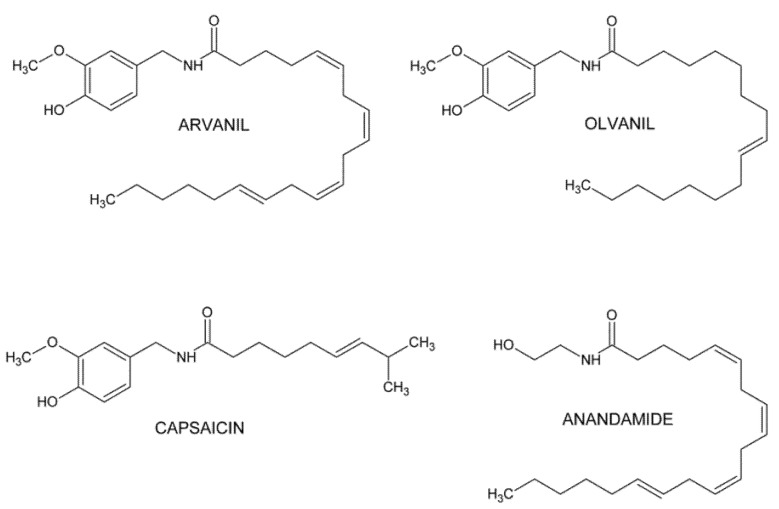
Chemical structure of arvanil and olvanil.

Arvanil and olvanil are structural “hybrids” because they present features of both groups as they express structural features of anandamide and capsaicin (Figure 1). Arvanil and olvanil belong to vanillyl-derivatives of anandamide and are non-pungent long-chain capsaicin analogs (non-pungent agonists of the TRPV1 receptor) and are potent agonists of TRPV1 and weak agonists of the CB1 receptor [6,7,8,9,10]. Arvanil is a more potent CB1 receptor ligand (*K*_i_ = 0.25–2.6 μM) than olvanil (*K*_i_ = 1.6 μM); moreover, it is a stronger inhibitor of the anandamide membrane transporter than olvanil (IC_50_ = 3.6 μM vs. IC_50_ = 9 μM) [10,11,12]. Some authors have claimed that both arvanil and olvanil are fatty acid amide hydrolase (FAAH) inhibitors rather than anandamide transport inhibitors [13]. The last property of both compounds may increase the amount of anandamide potentially available for cannabinoid and vanilloid receptor activation [10,12,14].

Lipophilicity of the TPRV1 agonists is directly related to the kinetics of TRPV1 activation and their pungency, but not to their potency. Arvanil is more pungent than olvanil [15,16]. Arvanil injection in animals evokes hypertension, decrease of respiratory rate, increase of tidal volume, and diaphragm activity. Fall in respiratory rate and increase of diaphragm activity are mediated by vanilloid VR1 receptors; rise of tidal volume is mediated by both VR1 and CB1 receptors, but hypertension might depend on different mechanisms [17]. In contrast, subcutaneous administration of olvanil (in doses of 0.05–5 mg/kg) does not produce adverse effects on cardiac parameters (heart rate, blood pressure), temperature, or spontaneous behaviors in mice [18]. Topical application of arvanil or olvanil produces vasodilatation and burning pain in humans [19]. Cisplatin (CDDP) is a widely used chemotherapeutic anticancer drug, exerting its action mainly by crosslinking with the purine bases during replication of DNA and activating several signal transduction pathways (including MAPK, ATR, p53, and p73) [20,21]. As a result of the anticancer activity, it causes DNA damage, blocks cell division, and induces apoptosis of replicating cells or necrosis. Due to development of cellular resistance mechanisms in cancer cells to CDDP, the drug achieves the best therapeutic outcomes when administered in combination with other agents in the treatment of various tumors [20,22].

Mitoxantrone (MTX), an anthracenedione antineoplastic agent, manifests its cytotoxic properties by inhibiting catalytic activity of topoisomerase II (the group of enzymes regulating replication and transcription of DNA) and intercalating to DNA and causing drug-stabilized cleavage complexes [23]. As a result of both mechanisms, MTX disrupts DNA synthesis and DNA repair. Additionally, MTX causes DNA aggregation and probably inhibits forming the microtubules [23,24].

Since molecular mechanisms of the action of arvanil and olvanil differ from the molecular mechanisms of the CDDP and MTX, this study was designed to evaluate the two drug combinations of arvanil with CDDP or MTX and olvanil with CDDP or MTX and compare the results to find out which of the tested hybrids could be more effective and favorable in the treatment of melanoma. The main hypothesis was that these drugs exert synergistic anti-proliferative interactions in the primary and metastatic melanoma cell lines (A375, SK–MEL 28, FM55P, and FM55M2).

To determine the types of interactions between arvanil/olvanil and CDDP or MTX, isobolographic analysis was performed, which is considered to be the gold standard in the evaluation of types of drug–drug interactions in cancer studies [25,26]. Moreover, in order to check the safety of the tested compounds, the impact of arvanil and olvanil on normal human keratinocytes and melanocytes was determined.

## 2. Results

**Figure 2:**
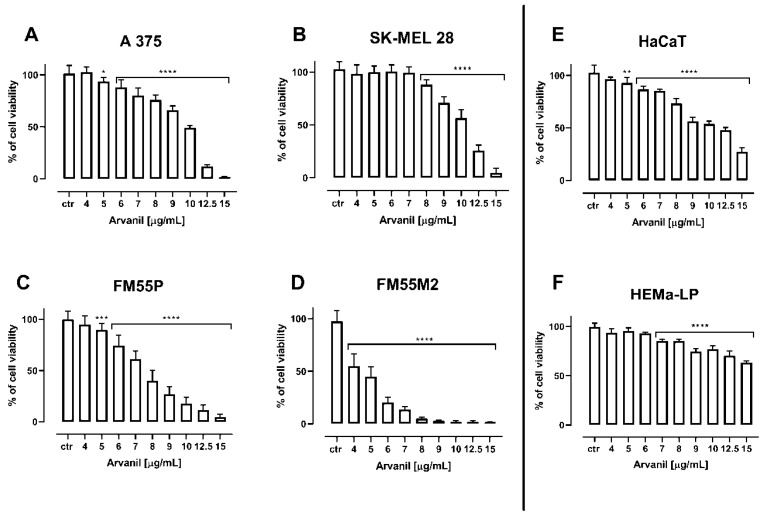
The impact of arvanil on the viability of malignant melanoma cell lines [A375 (**A**), SK–MEL 28 (**B**), FM55P (**C**) and FM55M2 (**D**)], normal human keratinocytes [HaCaT] (**E**), and normal human melanocytes [HEMa–LP] (**F**), measured by means of MTT assay after 72 h. Columns represent mean ± SEM (* p < 0.05, ** p < 0.01, *** *p* < 0.001, and **** *p* < 0.0001).

**Figure 3:**
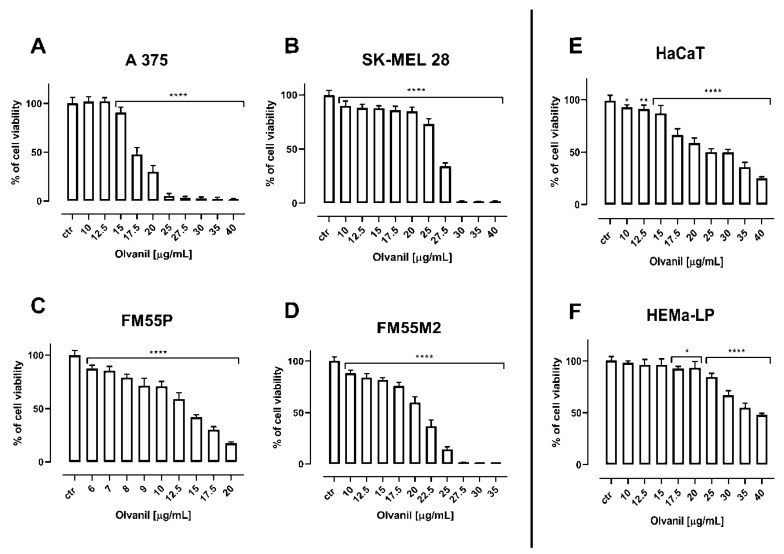
The impact of olvanil on the viability of malignant melanoma cell lines [A375 (**A**), SK–MEL 28 (**B**), FM55P (**C**) and FM55M2 (**D**)], normal human keratinocytes [HaCaT] (**E**), and normal human melanocytes [HEMa–LP] (**F**), measured by means of MTT assay. Columns represent mean ± SEM (* *p* < 0.05, ** *p* < 0.01, and **** *p* < 0.0001).

Arvanil and olvanil reduced the viability of various human melanoma cell lines (i.e., A375, SK–MEL 28, FM55P, and FM55M2) in a concentration-dependent manner, when applied separately (Figure 2 and Figure 3, Table 1). It is noteworthy that none of the solvents used in the respective control groups (i.e., ethanol, phosphate buffered saline (PBS), dimethyl sulfoxide (DMSO)) tested in relevant concentrations affected the viability of melanoma cells. The experimentally derived median inhibitory concentration (IC_50_) values for arvanil and olvanil in various melanoma cell lines are presented in Table 1. The experimentally derived IC_50_ values for CDDP and MTX in various malignant melanoma cell lines have been determined earlier [27].

**Figure 4:**
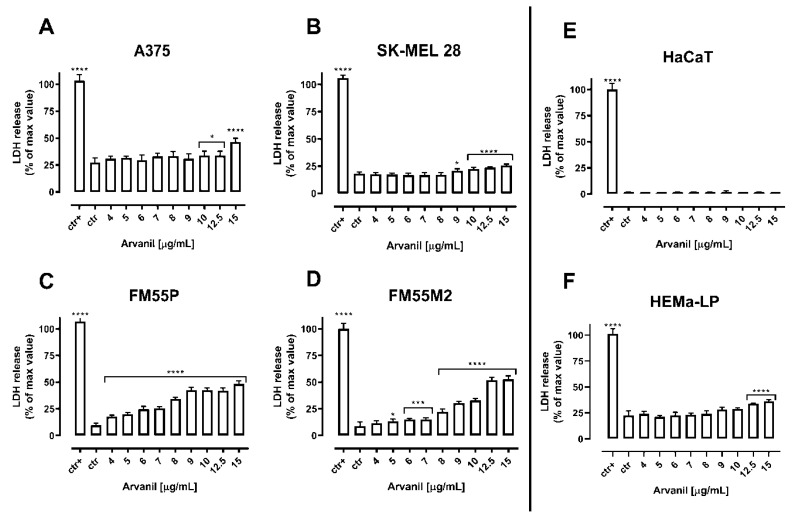
Cytotoxicity of arvanil to malignant melanoma cells [A375 (**A**), SK–MEL 28 (**B**), FM55P (**C**) and FM55M2 (**D**)], normal human keratinocytes [HaCaT] (**E**), and normal human melanocytes [HEMa–LP] (**F**). Lactate dehydrogenase ELISA kit was used to quantify cytotoxicity by measuring LDH activity released from damaged cells. Normal keratinocyte cells and melanoma cells were incubated for 72 h alone or in the presence of arvanil (4–15 µg/mL). The results are presented as the percentage in LDH released to the medium by treated cells versus cells grown in control medium (ctr) and cells treated with lysis buffer (ctr+). Data are presented as mean ± SEM (* *p* < 0.05, *** *p* < 0.001, and **** *p* < 0.0001).

**Figure 5:**
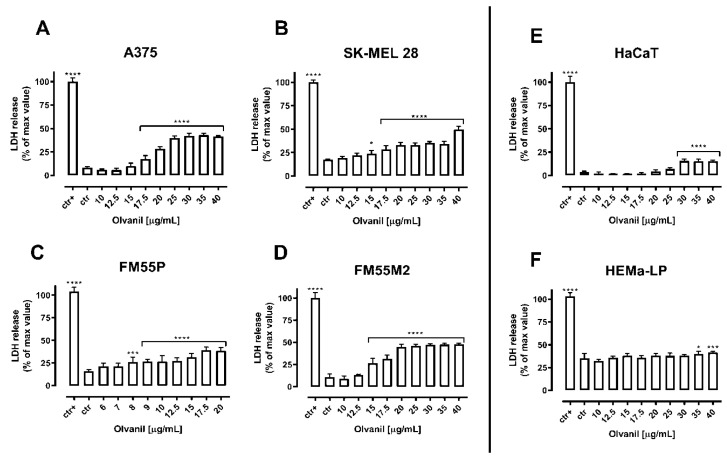
Cytotoxicity of olvanil to malignant melanoma cells [A375 (**A**), SK–MEL 28 (**B**), FM55P (**C**) and FM55M2 (**D**)], normal human keratinocytes [HaCaT] (**E**), and normal human melanocytes [HEMa–LP] (**F**). Lactate dehydrogenase ELISA kit was used to quantify cytotoxicity by measuring LDH activity released from damaged cells. Normal keratinocyte cells, normal human melanocytes, and melanoma cells were incubated for 72 h alone or in the presence of olvanil (10–40 µg/mL). The results are presented as the percentage in LDH released to the medium by treated cells versus cells grown in control medium (ctr) and cells treated with lysis buffer (ctr+). Data are presented as mean ± SEM (* *p* < 0.05, *** *p* < 0.001, and **** *p* < 0.0001).

The quantification of the cytotoxicity of both arvanil and olvanil in malignant melanoma cell lines, normal keratinocytes, and normal human melanocytes was performed in the LDH test. The diagrams show that the cytotoxicity of arvanil and olvanil in various malignant melanoma cell lines grows in a concentration-dependent manner while they present no significant impact on normal human keratinocytes and melanocytes (Figure 4 and Figure 5).

**Figure 6:**
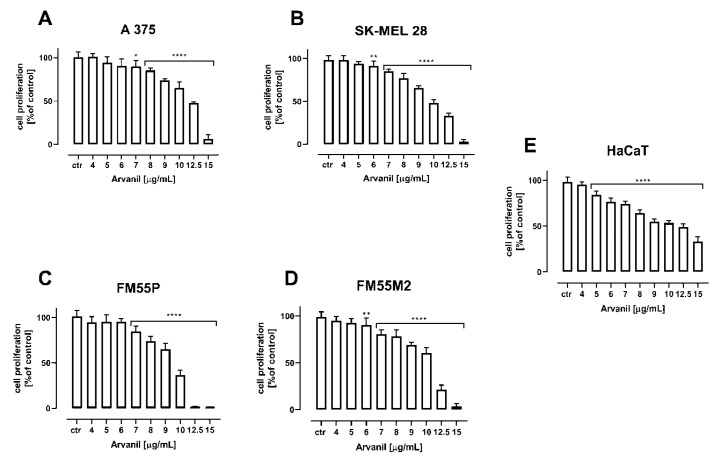
The effect of arvanil on the proliferation of malignant melanoma cell lines [A375 (**A**), SK–MEL 28 (**B**), FM55P (**C**), FM55M2 (**D**)], and normal human keratinocytes [HaCaT] (**E**) measured by means of BrdU assay after 72 h. Results are presented as mean ± SEM (* *p* < 0.05, ** *p* < 0.01, **** *p* < 0.0001).

**Figure 7:**
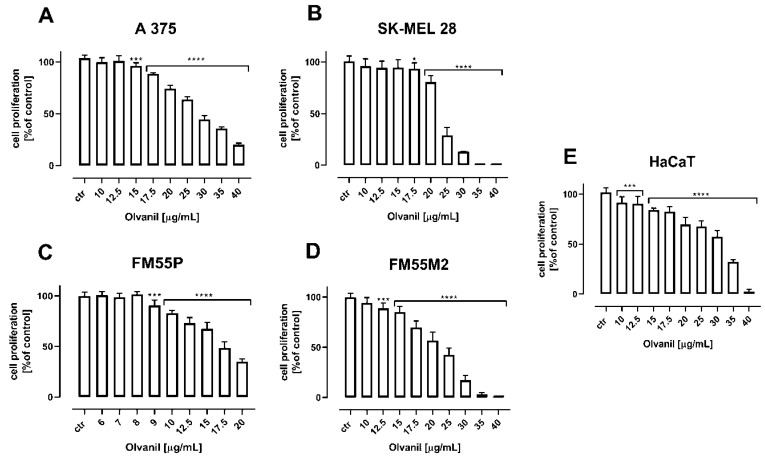
The effect of olvanil on the proliferation of malignant melanoma cell lines [A375 (**A**), SK–MEL 28 (**B**), FM55P (**C**), FM55M2 (**D**)], and normal human keratinocytes [HaCaT] (**E**) measured by means of BrdU assay after 72 h. Results are presented as mean ± SEM (* *p* < 0.05, *** *p* < 0.001, **** *p* < 0.0001).

In the BrdU test, both arvanil and olvanil inhibited the proliferation of all tested melanoma cell lines (A375, SK–MEL 28, FM55P, and FM55M2) and normal human keratinocytes (HaCaT) (Figure 6 and Figure 7). Arvanil at a concentration of 12.5 µg/mL and higher inhibited the proliferation of normal human keratinocytes (HaCaT) in approx. 50% (Figure 6). Olvanil produces the same effect in concentrations exceeding 30 µg/mL (Figure 7).

**Figure 8:**
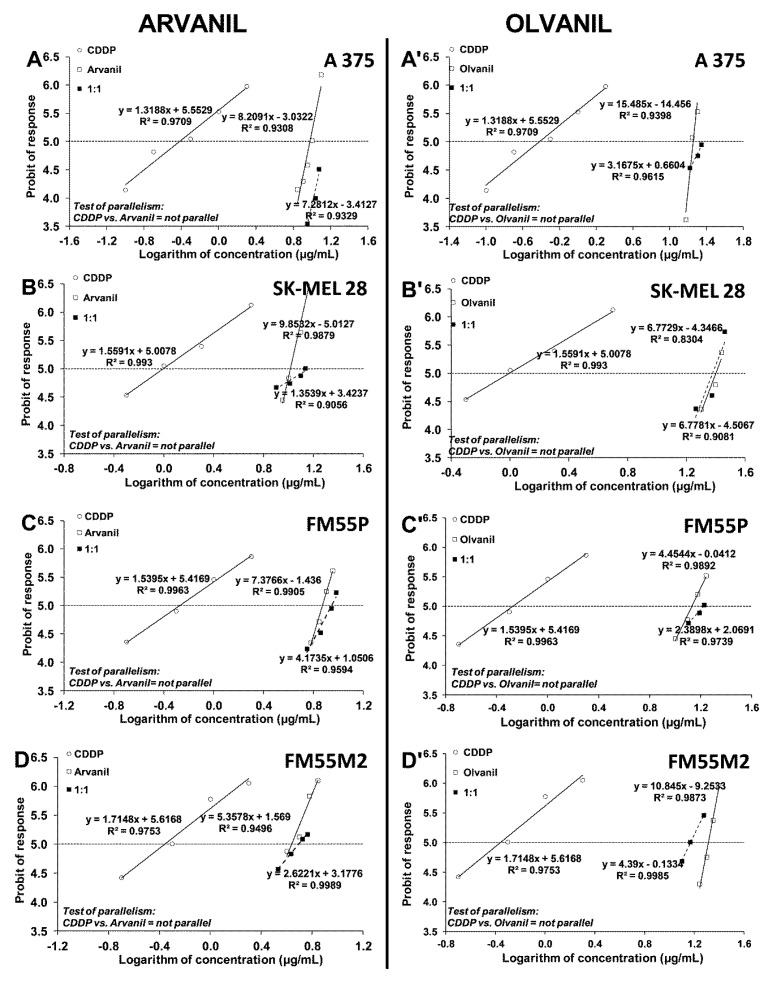
Concentration–effect lines for arvanil, olvanil, and CDDP administered alone and in combination in the fixed-ratio of 1:1, illustrating the anti-proliferative effects of the drugs in the malignant melanoma cell lines: A375 (**A**,**A’**), SK–MEL 28 (**B**,**B’**), FM55P (**C**,**C’**), and FM55M2 (**D**,**D’**) measured in vitro by the MTT assay. Test for parallelism confirmed that the experimentally determined concentration–effect lines for arvanil, olvanil, and CDDP (administered alone) are mutually non-parallel to each other in A375, SK–MEL 28, FM55P, and FM55M2 cell lines.

**Figure 9:**
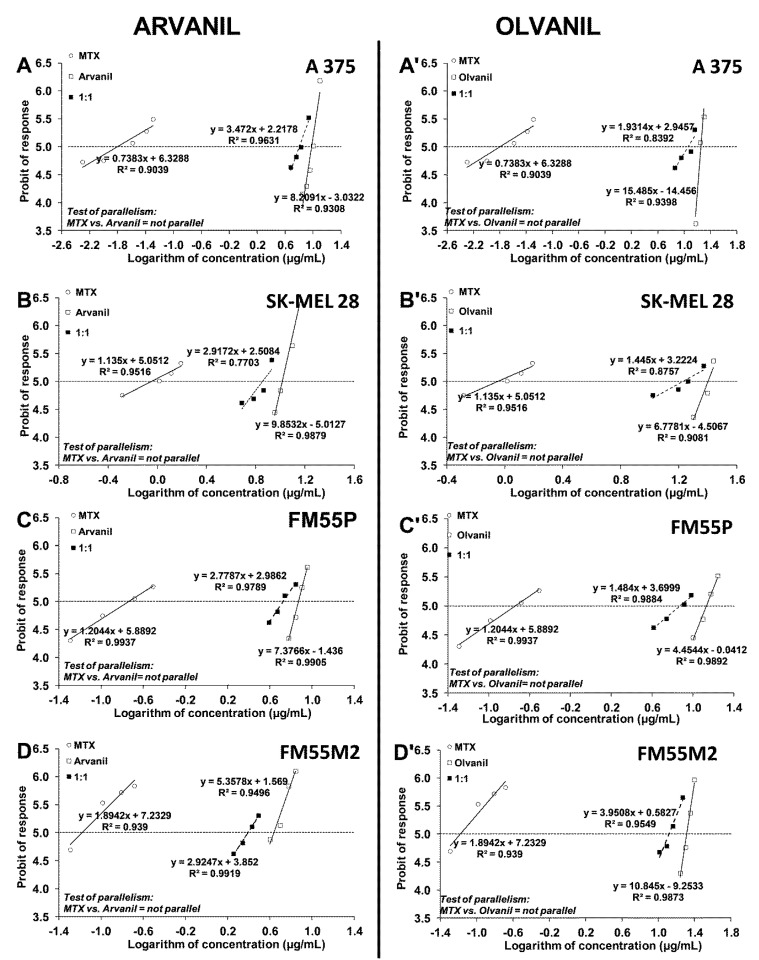
Concentration–effect lines for arvanil, olvanil, and MTX administered alone and in combination in the fixed-ratio of 1:1, illustrating the anti-proliferative effects of the drugs in the malignant melanoma cell lines: A375 (**A**,**A’**), SK–MEL 28 (**B**,**B’**), FM55P (**C**,**C’**), and FM55M2 (**D**,**D’**) measured in vitro by the MTT assay. Test for parallelism confirmed that the experimentally determined concentration–effect lines for arvanil, olvanil, and MTX (administered alone) are mutually non-parallel to each other in A375, SK–MEL 28, FM55P, and FM55M2 cell lines.

After calculating the IC_50_ values for arvanil and olvanil when used separately, mixtures of the two cannabinoid ligands with either CDDP or MTX (at a fixed drug dose ratio of 1:1) in the melanoma cell lines (A375, SK–MEL 28, FM55P, and FM55M2) were examined in the MTT assay. All the mentioned cell lines were incubated with different increasing concentrations of arvanil or olvanil and CDDP or MTX. The log-probit analysis of concentration-response anti-proliferative effect produced by the respective two-drug mixtures allowed the calculation of the experimentally derived IC_50mix_ values for the combinations studied in the MTT assay. The obtained results presented the concentration-dependent reduction in cancer cell viability (Figure 8 and Figure 9). The test of parallelism between the concentration-response lines for the studied drugs (arvanil + CDDP, arvanil + MTX, olvanil + CDDP, and olvanil + MTX) revealed that all the lines are not collateral to each other in various melanoma cell lines (Figure 8 and Figure 9).

**Figure 10:**
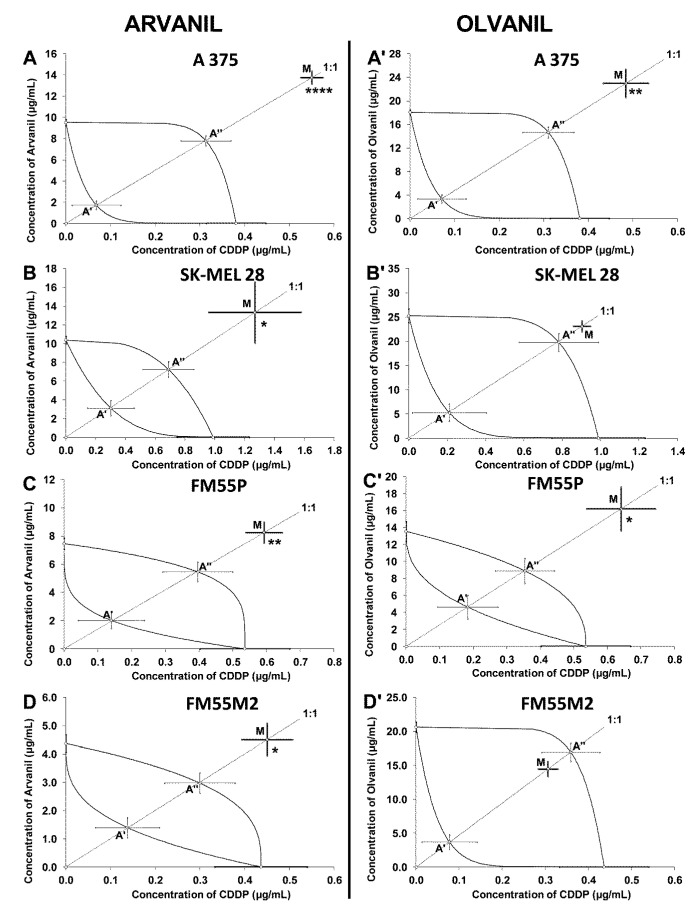
Isobolograms showing interactions between arvanil, olvanil, and cisplatin (CDDP) with respect to their anti-proliferative effects on A375 (**A**,**A’**), SK–MEL 28 (**B**,**B’**), FM55P (**C**,**C’**), and FM55M2 (**D**,**D’**) malignant melanoma cell lines measured in vitro by the MTT assay. Points **A’** and **A”** depict the theoretically calculated IC_50add_ values for both lower and upper isoboles of additivity, respectively. The points M represent the experimentally derived IC_50mix_ values for total concentration of the mixture of arvanil or olvanil with CDDP that produced a 50% anti-proliferative effect in malignant melanoma cell lines measured in vitro by the MTT assay. * *p* < 0.05, ** *p* < 0.01, and **** *p* < 0.0001 vs. the respective IC_50add_ value.

Isobolographic analysis of interactions for non-parallel concentration-response lines revealed that the combination of arvanil with CDDP at the fixed ratio of 1:1 exerted antagonistic interactions in the A375 melanoma cell line (Figure 10A). The IC_50mix_ value for this combination significantly differed from the upper IC_50add_ value (Student’s *t*-test with Welch correction—*t* = 7.46; df = 228.2; *p <* 0.0001; Table 2). On the isobologram, the IC_50mix_ for the mixture of arvanil + CDDP was placed drastically above the upper line of additivity for the A375 melanoma cell line, indicating an antagonistic interaction between the drugs (Figure 10A). The antagonistic interaction was also observed for the mixture of arvanil with CDDP at the fixed ratio of 1:1 in the SK–MEL 28 cell line (Figure 10B). In this case, the IC_50mix_ value for this combination significantly differed from the upper IC_50add_ value (Student’s *t*-test with Welch correction—*t* = 2.06; df = 115.1; *p =* 0.041; Table 2). On the isobologram, the IC_50mix_ value for the combination of arvanil + CDDP was graphically depicted above the upper line of additivity for the SK–MEL 28 melanoma cell line, indicating an antagonistic interaction between the drugs (Figure 10B). The combination of arvanil with CDDP at the fixed ratio of 1:1 exerted antagonistic interactions in the FM55P cell line (Figure 10C). The IC_50mix_ value for the combination of arvanil + CDDP significantly differed from the upper IC_50add_ value (Student’s *t*-test with Welch correction—*t* = 2.64; df = 184.7; *p =* 0.0089; Table 2). On the isobologram, the IC_50mix_ value for the combination of arvanil + CDDP was graphically plotted significantly above the upper line of additivity for the FM55P melanoma cell line, indicating an antagonistic interaction between the drugs (Figure 10C). Similarly, the combination of arvanil + CDDP (fixed ratio of 1:1) tested in the FM55M2 melanoma cell line exerted antagonistic interactions (Figure 10D). The IC_50mix_ value for the combination of arvanil + CDDP in the FM55M2 cell line significantly differed from the upper IC_50add_ value (Student’s *t*-test with Welch correction—*t* = 2.20; df = 179.1; *p =* 0.0291; Table 2). On the isobologram, the IC_50mix_ value for the combination of arvanil + CDDP was placed significantly above the upper line of additivity for the FM55M2 melanoma cell line, indicating an antagonistic interaction between the drugs (Figure 10D).

With the isobolographic analysis of interactions for non-parallel concentration-response lines, it was revealed that the combination of olvanil with CDDP (at the fixed ratio of 1:1) exerted antagonistic interactions in the A375 cell line (Figure 10A’). The IC_50mix_ value for this combination significantly differed from the upper IC_50add_ value (Student’s *t*-test with Welch correction—*t* = 3.22; df = 124.9; *p =* 0.0016; Table 2). On the isobologram, the IC_50mix_ for the mixture of olvanil + CDDP was placed considerably above the upper line of additivity for the A375 melanoma cell line, indicating an antagonistic interaction between the drugs (Figure 10A’). In contrast, an additive interaction with a slight tendency towards antagonism was observed for the mixture of olvanil with CDDP at the fixed ratio of 1:1 in the SK–MEL 28 cell line (Figure 10B’). In this case, the IC_50mix_ value for this combination did not differ from the upper IC_50add_ value (Student’s *t*-test with Welch correction—*t* = 1.46; df = 213.0; *p =* 0.145; Table 2). On the isobologram, the IC_50mix_ value for the combination of olvanil + CDDP was graphically depicted close to the upper line of additivity for the SK-MEL28 melanoma cell line, indicating an additive interaction between the drugs (Figure 10B’). The combination of olvanil with CDDP at the fixed ratio of 1:1 exerted antagonistic interactions in the FM55P cell line (Figure 10C’). The IC_50mix_ value for the combination of olvanil + CDDP significantly differed from the upper IC_50add_ value (Student’s *t*-test with Welch correction—*t* = 2.44; df = 119.4; *p =* 0.016; Table 2). On the isobologram, the IC_50mix_ value for the combination of olvanil + CDDP was plotted significantly above the upper line of additivity for the FM55P melanoma cell line, indicating an antagonistic interaction between the drugs (Figure 10C’). In contrast, the combination of olvanil + CDDP (fixed-ratio of 1:1) tested in the FM55M2 melanoma cell line exerted additive interactions (Figure 10D’). The IC_50mix_ value for the combination of olvanil + CDDP in the FM55M2 cell line did not differ from the upper IC_50add_ value (Student’s *t*-test with Welch correction—*t* = 1.41; df = 225.2; *p =* 0.161; Table 2). On the isobologram, the IC_50mix_ value for the combination of olvanil + CDDP was placed within the area bounded by two lines of additivity for the FM55M2 melanoma cell line, indicating an additive interaction between the drugs (Figure 10D’).

**Figure 11:**
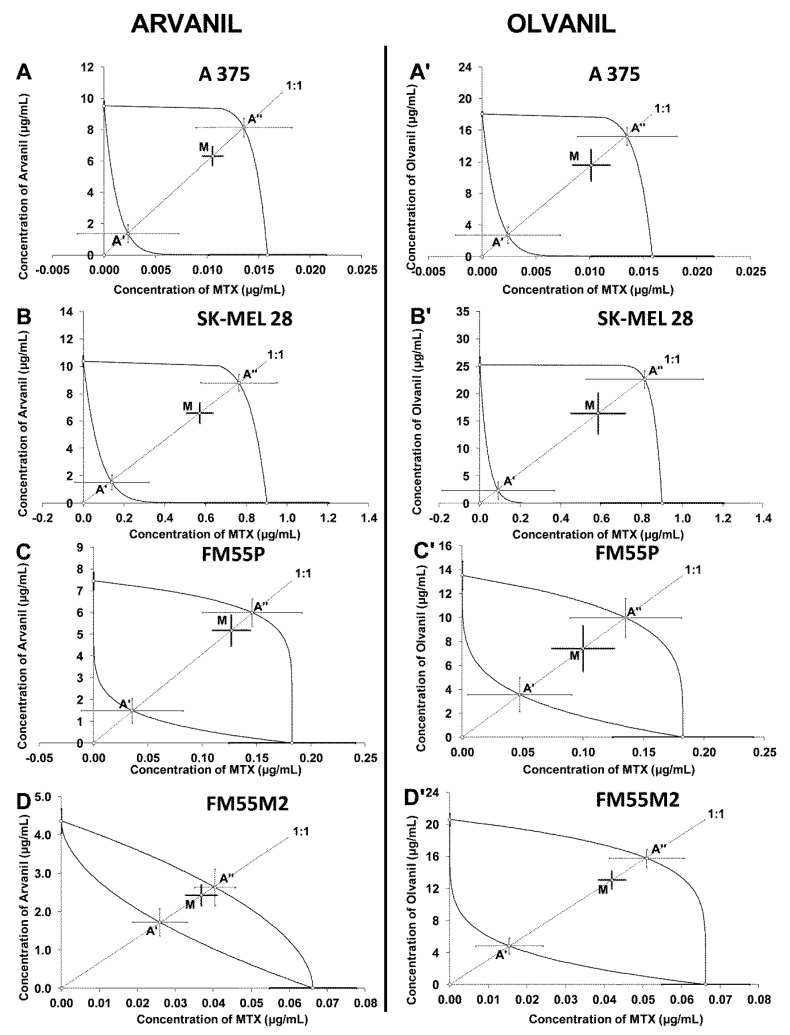
Isobolograms showing interactions between arvanil, olvanil, and mitoxantrone (MTX) with respect to their anti-proliferative effects on A375 (**A**,**A’**), SK–MEL 28 (**B**,**B’**), FM55P (**C**,**C’**), and FM55M2 (**D**,**D’**) malignant melanoma cell lines measured in vitro by the MTT assay. Points **A’** and **A”** depict the theoretically calculated IC_50add_ values for both lower and upper isoboles of additivity, respectively. The points M represent the experimentally derived IC_50mix_ values for total concentration of the mixture of arvanil or olvanil with MTX that produced a 50% anti-proliferative effect in malignant melanoma cell lines measured in vitro by the MTT assay.

For the combinations of arvanil with MTX, it was isobolographically found that the combination of arvanil with MTX at the fixed ratio of 1:1 exerted additive interactions in the A375 melanoma cell line (Figure 11A). The IC_50mix_ value for this combination did not differ from the upper IC_50add_ value (Student’s *t*-test with Welch correction—*t* = 1.83; df = 205.6; *p =* 0.069; Table 2). On the isobologram, the IC_50mix_ for the mixture of arvanil + MTX was placed within the area bounded by two isoboles of additivity for the A375 melanoma cell line, indicating an additive interaction between the drugs (Figure 11A). An additive interaction was also observed for the mixture of arvanil with MTX at the fixed ratio of 1:1 in the SK–MEL 28 cell line (Figure 11B). In this case, the IC_50mix_ value for this combination did not differ from the IC_50add_ value (Student’s *t*-test with Welch correction—*t* = 1.92; df = 233.9; *p =* 0.056; Table 2). On the isobologram, the IC_50mix_ value for the combination of arvanil + MTX was depicted within the area of additivity for the SK-MEL28 melanoma cell line, illustrating an additive interaction between the drugs (Figure 11B). The combination of arvanil with MTX (1:1) exerted additive interactions in the FM55P cell line (Figure 11C). The IC_50mix_ value for the combination of arvanil + MTX did not differ from the IC_50add_ value (Student’s *t*-test with Welch correction—*t* = 0.84; df = 176.9; *p =* 0.404; Table 2). On the isobologram, the IC_50mix_ value for the combination of arvanil + MTX was graphically plotted within the area bounded by two lines of additivity for the FM55P melanoma cell line, indicating an additive interaction between the drugs (Figure 11C). The combination of arvanil + MTX (1:1) tested in the FM55M2 melanoma cell line exerted additive interactions (Figure 11D). The IC_50mix_ value for the combination of arvanil + MTX in the FM55M2 cell line did not differ from the IC_50add_ value (Student’s *t*-test with Welch correction—*t* = 0.38; df = 249.4; *p =* 0.707; Table 2). On the isobologram, the IC_50mix_ value for the combination of arvanil + MTX was placed within the area bounded by two isoboles of additivity for the FM55M2 melanoma cell line, indicating an additive interaction between the drugs (Figure 11D).

Similarly, for the mixtures of olvanil with MTX, it was found that the combination of olvanil with MTX (at the fixed ratio of 1:1) exerted additive interactions in the A375 cell line (Figure 11A’). The IC_50mix_ value for this combination did not differ from the IC_50add_ value (Student’s *t*-test with Welch correction—*t* = 1.60; df = 158.5; *p =* 0.111; Table 2). On the isobologram, the IC_50mix_ for the mixture of olvanil + MTX was placed between two isoboles of additivity for the A375 melanoma cell line, indicating an additive interaction between the drugs (Figure 11A’). An additive interaction was also observed for the mixture of olvanil with MTX at the fixed ratio of 1:1 in the SK–MEL 28 cell line (Figure 11B’). In this case, the IC_50mix_ value for this combination did not differ from the IC_50add_ value (Student’s *t*-test with Welch correction—*t* = 1.50; df = 136.9; *p =* 0.136; Table 2). On the isobologram, the IC_50mix_ value for the combination of olvanil + MTX was graphically depicted within the area bounded by two isoboles of additivity for the SK–MEL 28 melanoma cell line, indicating an additive interaction between the drugs (Figure 11B’). The combination of olvanil with MTX (1:1) exerted additive interactions in the FM55P cell line (Figure 11C’). The IC_50mix_ value for the combination of olvanil + MTX did not differ from the IC_50add_ value (Student’s *t*-test with Welch correction—*t* = 1.02; df = 166.0; *p =* 0.308; Table 2). On the isobologram, the IC_50mix_ value for the combination of olvanil + MTX was plotted between two lines of additivity for the FM55P melanoma cell line, indicating an additive interaction between olvanil and MTX (Figure 11C’). The combination of olvanil + MTX (1:1) tested in the FM55M2 melanoma cell line exerted additive interactions (Figure 11D’). The IC_50mix_ value for the combination of olvanil + MTX in the FM55M2 cell line did not differ from the IC_50add_ value (Student’s *t*-test with Welch correction—*t* = 1.73; df = 242.2; *p =* 0.085; Table 2). On the isobologram, the IC_50mix_ value for the combination of olvanil + MTX was placed within the area bounded by two lines of additivity for the FM55M2 melanoma cell line, indicating an additive interaction between the drugs (Figure 11D’).

## 3. Discussion

Results from this study confirmed that both arvanil and olvanil inhibited, in a concentration dependent manner, the proliferation of malignant melanoma cell lines. Both arvanil and olvanil when combined with CDDP produced antagonistic interactions in the MTT assay, whereas the combinations of arvanil and olvanil with MTX exerted additive interactions in various melanoma cell lines. Both cannabinoid ligands exerted quite similar types of interactions in the in vitro MTT assay, when combined with CDDP and MTX, as that of cannabidiol in various melanoma cell lines [27].

Previously, it was observed that arvanil caused TRPV1-dependent Ca^2+^ response and cell death in high-grade astrocytoma in vitro. In an animal model of astrocytoma, it reduced tumor growth and prolonged life of the experimental mice. A suggested mechanism of arvanil’s antineoplastic activity was the stimulation of the endoplasmic reticulum stress pathway by activating transcription factor-3 (ATF3) [28]. Moreover, arvanil has been reported to cause cell death of primary prostate cancer cell line (PPC-1) and metastatic (TSU) line; however, the mechanism of the cell death has not been determined [29]. Arvanil also induced apoptosis in the lymphoid Jurkat T-cell line, while it does not affect primary peripheral blood T lymphocytes. The apoptosis of lymphoid cells induced by arvanil was TRPV1 and CB-independent and involved the signaling complex and the activation of caspase-8, independently of any distinct phase of cell cycle [30,31].

Arvanil and olvanil present anti-invasive activity, which is not dependent on CB1 and TRPV receptors in the human small-cell lung cancer (DMS 114 and DSM 53) cell lines. The mechanism of this favorable effect was mediated by the activation of the MAPK pathway [32]. Arvanil and olvanil also exert anti-proliferative effects in human breast cancer cells MCF-7 and T-47D by suppressing the prolactin receptor- and/or nerve growth factor (NGF)-induced cell proliferation via activation of the CB1 receptor that leads to the inhibition of adenylyl cyclase and stimulation of the MAPK pathway [33]. A recent study on human lung cancer cell lines showed that arvanil combined with irinotecan produced synergistic interactions in H69-CPR and PC9-CDDP, i.e., cisplatin-resistant lung cancer cells [34].

In the case of olvanil, it inhibited human breast cancer cell line EFM-19 proliferation and its anti-proliferative action was enhanced by palmitoylethanolamide (PEA). It was suggested that the possible mechanism of this phenomenon may be related to the potentiation of activity of VR1 receptors [35]. Olvanil has been reported to reduce numbers of breast cancer metastases to lung and liver. The main mechanism involved in this action was activation of neuro-immune pathways via stimulation of TRPV1-containing sensory nerve fibers, causing cytokine response and increase of T-cell count [36]. The above-mentioned facts indicate that the anti-viability effects of arvanil and olvanil are not cancer-specific since both compounds inhibited proliferation in various cancer cell lines.

Comparison of effects produced by the two cannabinoid and vanilloid receptor ligands (i.e., arvanil and olvanil) in this study revealed that the more efficacious agent in relation to the anti-proliferative effect was arvanil due to its lower IC_50_ values in all the tested in vitro cell lines. However, it was found that olvanil exerted more favorable effects when combined with CDDP in two melanoma cell lines (i.e., SK–MEL 28 and FM55M2) than arvanil did. In this case, arvanil produced antagonistic interactions when combined with CDDP, while olvanil exerted additive interactions when combined with CDDP in these two cell lines. The observed difference remains unexplained at present. Since the molecular mechanisms of action of both ligands are quite similar, the exerted interaction should also be similar. However, some differences observed in in vitro studies must be explained as a result of activation of different pathways involved in the anti-proliferative effects of CDDP and arvanil or olvanil. Of note, some differences in the potency of arvanil and olvanil to both CB1 and VR1 receptors should be kept in mind while explaining the observed effects in these two cell lines (SK–MEL 28 and FM55M2). Another fact deserves more attention. In primary melanoma cell lines (A375 and FM55P), olvanil exerted antagonistic interactions, whereas in the metastatic cell lines (SK–MEL 28 and FM55M2), olvanil produced additive interactions when combined with CDDP. Perhaps more advanced studies will shed light on these combinations. Since antagonistic interactions between drugs are not favorable during anti-cancer treatments, arvanil should not be combined with CDDP. From a pharmacological viewpoint, one drug inactivates the second one, and finally, the resulting combination has an antagonistic nature. In contrast, the combinations of MTX with both ligands (arvanil and olvanil) deserve a pre-clinical recommendation as favorable combinations during anti-cancer treatments.

As mentioned in the introduction, the first line of treatment of melanoma is based on surgical excision of the neoplasm. However, the application of multidrug therapy can be considered when there exists a high risk of appearance of neoplastic transformation in various places or when metastatic foci are expected to be present. In such cases, multidrug treatment should slow down the progression of melanoma. This is the reason to pre-clinically test various combination of arvanil and olvanil with CDDP and MTX. A general rule in oncology is to combine drugs producing the anticancer effects via different molecular mechanisms activating various pathways so the drugs could mutually cooperate in inhibiting the proliferation of melanoma cells.

Additionally, various cannabinoids may improve quality of life for oncologic patients, including these who suffer from melanoma [37]. One of the most burdensome side effects of anticancer pharmacotherapy is emesis. For example, it occurs in up to 90% of patients treated with cisplatin or dacarbazine [38]. Noteworthy, both CB1 and TPRV agonists suppress nausea and vomiting by acting on the brainstem nuclei [39]. In animal studies, olvanil at a dose of 5 mg/kg reduced CDDP-induced emesis, but only during the acute phase, lasting for 24 h [18]. In the delayed phase, lasting for 2–3 days, it neither reduced emesis nor reduced food and water intake, but it seemed to potentiate the reduction of food consumption [40].

Arvanil and olvanil also produce an anti-nociceptive effect that is independent on CB1 or TPRV1 receptor activation [6,8,41,42]. The main suggested mechanism of action of olvanil is inhibition of voltage-activated Ca^2+^ channels through a biochemical pathway that involves intracellular Ca^2+^-calmodulin and calcineurin, therefore producing desensitization of nociceptors in nociceptive neurons [42]. Of note, the antinociceptive properties of both ligands may be advantageous for improving the quality of life of patients.

## 4. Materials and Methods

### 4.1. Cell Lines

Two cell lines, A375 (primary malignant melanoma) and SK–MEL 28 (metastatic malignant melanoma), were purchased from the American Type Culture Collection (ATCC, Manassas, VA, USA) and cultured in Dulbecco’s Modified Eagle’s Medium—high glucose (DMEM) and Eagle’s minimal essential medium (EMEM) (Sigma-Aldrich, St. Louis, MA, USA), respectively. In contrast, another primary (FM55P) and metastatic (FM55M2) malignant melanoma cell lines were purchased from the European Collection of Cell Cultures (ECACC, Salisbury, UK) and cultured in RPMI—1640 Medium (Sigma-Aldrich). All culture media were supplemented with 10% Fetal Bovine Serum (FBS; Sigma-Aldrich) and 1% of penicillin/streptomycin (Sigma-Aldrich). Cultures were kept at 37 °C in a humidified atmosphere of 95% air and 5% CO_2_. The cells were grown to 80% confluence.

### 4.2. Drugs

Mitoxantrone (MTX—Sigma-Aldrich) was dissolved in DMSO as stock solutions. Cisplatin (CDDP—Sigma-Aldrich) was dissolved in phosphate buffered saline (PBS) with Ca^2+^ and Mg^2+^. The arvanil and olvanil (Tocris, Bristol, UK) were dissolved in ethanol as stock solutions in concentration of 5 mg/mL. The drugs were dissolved to the respective concentrations with culture medium before use.

### 4.3. Cell Viability Assessment

A375, SK–MEL 28, FM55P, and FM55M2 cells were placed on 96-well plates (Nunc, Roskilde, Denmark) at a density of 3 × 10^4^ cells/mL, 2 × 10^4^ cells/mL, 2 × 10^4^ cells/mL, and 2 × 10^4^ cells/mL, respectively. On the next day, the culture medium was removed and cells were exposed to serial dilutions of arvanil, olvanil, CDDP, and MTX in fresh culture medium. Cell viability was assessed after 72 h by means of the MTT test, in which the yellow tetrazolium salt (MTT) was metabolized by viable cells to purple formazan crystals. The 72-h incubation time is the average doubling time for all tested melanoma cell lines. In the case of the SK–MEL 28 line, doubling time is 17.5 h [43]; for the A375 line, this time is shorter 6–12 h [44], but in the case of the FM55P and FM55M2 lines, most of the experiments encountered have an incubation time of 72 h [27,45,46]. After 72 h of incubation, cells were incubated for 3 h in the MTT solution (5 mg/mL, Sigma-Aldrich). Formazan crystals were solubilized overnight in sodium dodecyl sulfate (SDS) buffer (10% SDS in 0.01 N HCl) and the product was determined spectrophotometrically by measuring absorbance at 570 nm wavelength using a microplate spectrophotometer (Ledetect 96, Labexim Products, Lengau, Austria). Each treatment was performed in triplicate and each experiment was repeated 3 times.

### 4.4. Cytotoxicity Assessment—LDH Assay

Optimized amounts of A375 (2 × 10^4^/mL), SK–MEL 28 (3 × 10^4^/mL), FM55P (2 × 10^4^/mL), FM55M2 (2 × 10^4^/mL), and normal human keratinocytes HaCaT (1 × 10^4^/mL) cells were placed on 96-well plates (Nunc). After 24 h, cells were washed in PBS, and then exposed to increasing concentrations of arvanil or olvanil in the proper fresh culture medium. The cytotoxicity was estimated by measuring cytoplasmic lactate dehydrogenase (LDH) activity released from damaged cells after exposure to arvanil or olvanil for 72 h. LDH assay was performed according to the manufacturer’s instructions (Cytotoxicity Detection KitPLUS LDH) (Roche Diagnostics, Mannheim, Germany). Concisely, after the collection of 50 µL of cell medium from each well, the 50 µL of reaction mixture (freshly prepared) was added and incubated for 30 min at RT. The next step was adding 25 µL of Stop solution to each well on the 96-well plate. Finally, absorbance was measured at two different wavelengths, one being the “measurement wavelength” (492 nm) and the other “reference wavelength” (690 nm) using a microplate spectrophotometer (Ledetect 96, Labexim Products, Lengau, Austria). Maximum LDH release (positive control) was achieved by the addition of lysis buffer to untreated control cells. The average values of the culture medium background were subtracted from all values of experimental wells. The percentage of death cells was calculated in relation to the maximum LDH release.

### 4.5. Cell Proliferation Assay

Cell Proliferation Elisa, BrdU Kit (Roche Diagnostics, Mannheim, Germany) was performed by following the manufacturer’s instructions. Optimized amounts of A375 (2 × 10^4^/mL), SK–MEL 28 (3 × 10^4^/mL), FM55P (2 × 10^4^/mL), and FM55M2 (2 × 10^4^/mL) cells were placed on a 96-well plate (Nunc) (100 μL/well). On the following day, the cancer cells were treated with increased concentrations of arvanil and olvanil for 48 h. After that, 10 µL/well BrdU Labeling Solution (100 µM) was added and cells were re-incubated for an additional 24 h at 37 °C. Then, the culture medium was removed and cells were fixed in FixDenat solution (200 µL/well) (30 min, at RT). The working solution of anti-BrdU antibody coupled with horseradish peroxidase (anti-BrdU-POD) were subsequently added (100 µL/well) (90 min, RT) and detected using tetramethylobenzidine substrate (TMB) (100 µL/well) (30 min, RT). To stop the enzymatic reaction, a total of 1 M (25 µL/well) sulfuric acid was added. The quantitation was performed spectrophotometrically at 450 nm using a microplate spectrophotometer (Ledetect 96, Labexim Products, Lengau, Austria).

### 4.6. Isobolographic Analysis of Interactions

Due to log-probit analysis, it was possible to transform percentage of inhibition of cell viability into probit and concentrations of arvanil and olvanil when administered singly (in the A375, SK–MEL 28, FM55P, and FM55M2 melanoma cell lines) into logarithm of concentrations as reported earlier [27]. Subsequently, from the log-probit concentration–response lines, the median inhibitory concentrations (IC_50_ values) of arvanil and olvanil were calculated [47]. Linear Loewe’s additivity model allowed us to verify the parallelism of probit-type concentration–response curves for the studied drugs (arvanil, olvanil) with CDDP or MTX, as reported earlier [47,48,49,50,51]. Verification of parallelism revealed that none of the tested two-drug combinations had their lines mutually collateral in all the tested cell lines in the in vitro MTT assay. In the type I isobolographic analysis for non-parallel concentration–response effect lines, the additivity is defined as an area bounded by two lower and upper isoboles of additivity [48,50,51,52,53,54,55]. After calculating the median additive inhibitory concentrations (IC_50add_) for the two-drug mixture of (arvanil or olvanil) with CDDP or MTX, which theoretically should inhibit 50% of cell viability, as demonstrated earlier [50], the experimentally derived IC_50mix_ (at the fixed ratio of 1:1) were determined in malignant melanoma cell lines measured in vitro by the MTT assay.

Because arvanil and olvanil have never been tested in melanoma cell lines (A375, SK–MEL 28, FM55P, and FM55M2), the selection of their concentrations in the MTT assay was purely empirical in this study. Although a few concentrations of the tested substances in the MTT assay were plotted graphically (Figure 2 and Figure 3), many more concentrations of the drugs were experimentally tested, but not illustrated graphically. Generally, the concentrations of the tested drugs in in vitro studies are selected in such a way that the observed effects illustrate a clear-cut dose-dependent reduction in cell viability [56,57]. Subsequently, the chosen concentrations of the drugs along with their anti-viability effects underwent the log-probit transformation, allowing the calculation of the IC_50_ values for the drugs. Of note, calculation of the IC_50_ from sigmoidal Hill and linear log-probit equations provide the same results and both methods are used equivalently in toxicological studies [27,55,58,59,60]. However, the main problem in in vitro studies is linked with a confirmation of hypothesis that the tested compounds (arvanil and olvanil) really affect only one type of receptors. In the case of arvanil and olvanil, the compounds are “hybrids”, and they are capable of acting simultaneously on various receptors, channels, and targets; furthermore, it is unknown whether these drugs do not activate other, unknown as of yet, pathways at the same time [15,61]. Of note, the log-probit method does not consider one unique mechanism of action of the tested drugs, but it reflects the observed final effects, even if these effects are a result of activation of several various possible mechanisms [49]. This was the reason to preferentially use the log-probit method in this study, instead of the method concerned with agonist-receptor-based pharmacodynamics, for which nonlinear Hill equations are specifically modified to the hypothetical situation [59,62,63,64]. Generally, figures illustrating log-probit lines along with their respective equations provide information about parallelism of these concentrations-response relationship lines between the tested compounds (when administered alone). From these equations, it is possible to calculate the exact concentrations for each drug tested, as well as concentrations for the two-drug mixture at the fixed-ratio of 1:1 (Figure 8 and Figure 9).

### 4.7. Statistical Analysis

The statistical analysis of data was performed by means of GraphPad Prism 8.0 Statistic Software. One-way analysis of variance (ANOVA test) for multiple comparisons followed by Tukey’s significance test was used. The data are expressed as the mean ± standard error (SEM) (* p < 0.05, ** p < 0.01, *** p < 0.001, and **** p < 0.0001). The experimentally derived IC_50mix_ values for the mixture of arvanil or olvanil with CDDP and arvanil or olvanil with MTX were statistically compared with their respective theoretically additive IC_50add_ values by the use of the unpaired Student’s *t*-test with Welch correction, as described earlier [49].

## 5. Conclusions

We have found, for the first time, that arvanil and olvanil possess anti-viability effects in various malignant melanoma cell lines. Isobolographic analysis of the interactions of both arvanil and olvanil with either CDDP or MTX provided evidence that the more favorable combinations were those with MTX. Antagonistic interactions of arvanil and olvanil with CDDP votes against their recommendation in further experimental and clinical studies. However, more favorable profiles for the combinations of olvanil with CDDP were observed in metastatic cell lines (SK–MEL 28, FM55M2), while in primary malignant melanoma cell lines (A 375, FM55P), the combination of olvanil with CDDP exerted antagonistic interactions. Further experiments are needed to elucidate differences in activity of combinations in primary and metastatic melanoma cell lines.

## Figures and Tables

**Table 1:** The anti-proliferative effects of arvanil and olvanil administered singly in human malignant melanoma cell lines measured in vitro by the MTT assay.

Cell Line	Arvanil (µg/mL)	Arvanil (µM)	Olvanil (µg/mL)	Olvanil (µM)
A375	9.52 ± 0.30	21.65 ± 0.68	18.05 ± 0.40	43.22 ± 0.96
SK–MEL28	10.38 ± 0.40	23.64 ± 0.91	25.27 ± 1.43	60.51 ± 3.42
FM55P	7.46 ± 0.39	16.97 ± 0.89	13.54 ± 1.17	32.42 ± 2.80
FM55M2	4.37 ± 0.31	9.94 ± 0.71	20.62 ± 0.73	49.37 ± 1.75

Data are median inhibitory concentrations (IC_50_) values in µg/mL or µM (± SEM).

**Table 2:** Isobolographic analysis of interactions between arvanil, olvanil, CDDP, and MTX (at the fixed ratio of 1:1) in various melanoma malignant cell lines.

Drug Combination	Cell Line	IC_50mix_ (μg/mL)	n_mix_	Lower IC_50add_ (μg/mL)	n_add_	Upper IC_50add_ (μg/mL)	Interaction
Arvanil + CDDP	A375	14.30 ± 0.65 ****	96	1.77 ± 0.44	356	8.09 ± 0.52	Antagonism
	SK–MEL 28	14.60 ± 3.08 *	96	3.42 ± 0.96	140	7.93 ± 1.00	Antagonism
	FM55P	8.84 ± 0.81 **	72	2.15 ± 0.66	140	5.84 ± 0.79	Antagonism
	FM55M2	4.96 ± 0.63 *	96	1.53 ± 0.42	132	3.27 ± 0.44	Antagonism
Olvanil + CDDP	A375	23.44 ± 2.46 **	96	3.42 ± 0.73	286	14.95 ± 0.96	Antagonism
	SK–MEL 28	23.99 ± 1.18	96	5.48 ± 1.93	140	20.56 ± 2.03	Additivity
	FM55P	16.84 ± 2.70 *	72	4.80 ± 1.48	140	9.25 ± 1.56	Antagonism
	FM55M2	14.77 ± 1.12	96	3.75 ± 1.12	132	17.28 ± 1.40	Additivity
Arvanil + MTX	A375	6.33 ± 0.81	96	1.37 ± 0.57	306	8.16 ± 0.59	Additivity
	SK–MEL 28	7.15 ± 0.81	96	1.66 ± 0.64	140	9.57 ± 0.96	Additivity
	FM55P	5.31 ± 0.73	72	1.53 ± 0.60	140	6.14 ± 0.67	Additivity
	FM55M2	2.47 ± 0.28	96	1.75 ± 0.36	168	2.68 ± 0.48	Additivity
Olvanil + MTX	A375	11.58 ± 1.99	96	2.72 ± 1.03	306	15.23 ± 1.11	Additivity
	SK–MEL 28	16.99 ± 3.90	96	2.42 ± 1.83	140	23.46 ± 1.83	Additivity
	FM55P	7.52 ± 1.94	72	3.61 ± 1.46	140	10.12 ± 1.65	Additivity
	FM55M2	13.12 ± 1.10	96	4.82 ± 1.00	168	15.82 ± 1.10	Additivity

The IC_50_ values (in µg/mL± SEM) for the mixture of arvanil with CDDP, arvanil with MTX, olvanil with CDDP, and olvanil with MTX were determined experimentally (IC_50mix_) in four melanoma malignant cell lines in the in vitro MTT assay. The IC_50add_ values were calculated from the lower and upper isoboles of additivity. The *n*_mix_—total number of items experimentally determined; *n*_add_—total number of items calculated for the additive two-drug mixture; * *p* < 0.05, ** *p* < 0.01, and **** *p* < 0.0001 vs. the respective upper IC_50add_ value.

## Data Availability

Data are contained within the article.
